# Supplementary material for: Risk factors and survival in patients with COVID-19 in northeastern Brazil
Source: PLoS One. 2022 Nov 28;17(11):e0278213. doi: 10.1371/journal.pone.0278213 (PMC9704671; doi:10.1371/journal.pone.0278213)
Supplement: Attachment — Submitted filename: Response to Reviewers.docx [file pone.0278213.s001.docx]

We would like to thank all considerations made by the reviewers. We clarified and modified the manuscript as suggested. We also appreciate the opportunity to send this new version for peer review.

Reviewer #1: This is an interesting work from colleagues from Brazil providing insights on epidemiology and risk factors of hospitalized COVID-19 patients.

I have several comments for authors to consider

1. Can you provide percentage of people who were discarded due to unavailability of outcomes? This is important so as to mitigate selection bias.

*A total of 365 patients were registered on the eCOVID platform. Due to delays in seeking hospital care, lack of information, and unavailability of tests in Brazil, some patients may have undergone the exam outside the ideal window for virus identification. These patients were treated for COVID-19 but only based on the evolution of the clinical picture. We included only those records of patients who had a positive RT-PCR test for COVID-19 (N=280; 76.7%) since this was considered the gold standard for diagnosing COVID-19 worldwide at the time of data collection.*

2. Data analysis: How was the multiple imputation performed? Which software/platform were used?

*Thank you for your question. All statistical analyses were conducted using the SPSS statistical software, version 22 (SPSS, Inc., Chicago, IL, USA). Missing data were managed using multiple imputations by replacing missing cells with random values from a statistical model based on distribution and underlying assumptions on the nature of the missing data. Five imputed datasets were created, and the final estimates were the average of different sets of imputed estimates and standard errors. The multiple imputation approach was chosen because it generates more accurate values than those generated by single imputation methods.*

*The above information has also been included in the revised text.*

3. Page 6: what do you mean by "with death. studied outcomes"??

*Thank you for the comment. This typo was corrected in the new version.*

4. Why was the predictive accuracy, sensitivity and ROC of the regression model not calculated? Did you also account for the multicollinearity while selecting for variables to be included in the multivariate model? Please provide details?

*Thank you for the suggestion. We have now provided the ROC curve of the regression model in Figure 2. The ROC curve showed a 0.84 predictive capacity for the computed regression model.*

*Regarding multicollinearity, this was not a problem in our analysis. We used the stepwise regression method to compute the model since it is used to provide an initial screening of variables within a large set of variables; thus, accounting for multicollinearity. We specified the predictors we would like to include, while the SPSS screened which factors contributed to predicting our dependent variable and excluded those who did not. Thus, we usually end up with fewer predictors than we specify. However, those that remain in the model tend to have solid and significant β-coefficients in the expected direction.*

5. The manuscript needs significant editing for language and comprehension. For example, what do you mean by, "the unfamiliarity of the population with signs and symptoms of COVID-19 may have delayed healthcare seeking" (page 12) - healthcare seeking?

*Thank you for your comment. The scientific writing of the manuscript was revised, and all inconsistencies were corrected.*

*The term “delayed healthcare seeking” was also corrected for “delayed* ***health care*** *seeking” (with a space) and maintained in the text because it is common in the literature (doi: 10.2147/HIV.S210977; doi: 10.1177/1049732315588083; doi: 10.4102/phcfm.v9i1.1378).*

6. Several aspects of the results have not been discussed. Please organise the discussion so as to highlight key points and provide supporting discussion from relevant literature previously reported, if available.

7. In the discussion, suggest also discussing briefly that previous studies have reported prexisting factors such as diabetes are also associated with worse outcomes in COVID-19 patients ((see a metaanalysis by Bradley et al J Diabetes 2022 PMID: 34939735)

8. In the Discussion, you may also provide some indications, based on the findings and experiences from this study, some strategies which may be useful for future. Obviously role of telemedicine may also be discussed including streamlining telemedicine care for both hospitalised and non-hospitalised patients (see PMID: 33796502)

*Thank you for comments 6, 7, and 8. Below you can find the changes made to improve the understanding of the discussion.*

*All key points of the results were included in paragraph 1: epidemiological aspects, comorbidities, signs and symptoms, and mortality.*

*To optimize the discussion, new information was added in paragraph 4 (page 11).*

*To answer comment 8, information was added in the last paragraph of the discussion. Telemedicine was not included as a strategy, given difficulties for implementation in Brazil and the low adherence of public health systems. Thus, this would not be a viable strategy from an economic point of view according to the current situation in the country.*

9. If possible, please also provide the ROC figure of the final prognostic model.

*Thank you for the comment. The ROC figure of the final regression model has been provided (Figure 2*).

Reviewer #2: Dear Author(s),

Thank you for your esteemed efforts in increasing our collective knowledge about the predictors (risk factors) of mortality in hospitalized COVID-19 patients.

Please consider the following points:

1. Abstract: the "background" subsection is so broad and does not convey the objective of the study. Please re-write it.

*Thanks for the comment. Adjustments were made to the abstract.*

2. Abstract: add explanation for "hospital LOS"

*Thank you for the comment. Information about the term was added on page 5 (Variables and data collection).*

3. "A transmissible disease (COVID-19) caused by the SARS-CoV-2 virus increased the number of severe acute respiratory syndrome (SARS) cases worldwide since December 2019."
This sentence is not linguistically sound. COVID-19 is not an abbreviation for "a transmissible disease". SARS-CoV-2 is not explained. SARS refers to SARS-CoV-1, so please do not use it here.

*Thank you for the comment. We made the necessary adjustments to improve the understanding of the text.*

4. Introduction: do not use expressions like "to date". Use actual dates to be specific.

*Thank you! The text regarding this epidemiological data was updated.*

5. "The United States of America and Brazil correspond to 71% of cumulative cases of COVID-19 in the Americas (more than 46 million cases)3." How this sentence is useful for your narrative?! US and Brazil are the most populous countries in the region, so it makes sense that they will have the highest number of cases/fatalities.

*Thank you! The text regarding this epidemiological data was updated.*

6. Why did you the age of 65 years old as cut-off point for analysis?

*Thank you for your comment. It is well documented that the risk of severe COVID-19 increases with age. Since the CDC stated that adults aged ≥ 65 years were at greater risk of COVID-19 complications and death (*[*https://www.cdc.gov/aging/covid19-guidance.html*](https://www.cdc.gov/aging/covid19-guidance.html)*), we have decided to use this cut-off point to stratify individuals into younger vs. older adults. Other studies have also used this age as a cut-off for different analyses, as discussed in the manuscript.*

7. How did you decide whether the patient was critically or moderately ill on admission? Which categorization scheme have you used? Please mention that in your Methods section.

*Thank you for the comment. The information was added in the “Variables and data collection” of the methods section (page 5).*

8. Age (p = 0.001), dyspnea (p = 0.003), and presence of other heart diseases (p =0.005) were different between groups."
It is unclear which groups do you refer to?

*Thank you. We explain the subdivision of groups in the second paragraph of the Data Analysis section (Survivors and Non-survivors) and also in the revised paragraph. The aforementioned analyzes were conducted using these two groups, as shown in Table 1.*

9. In Table 1; you did not add percentages of signs and symptoms. Please add them.

*Thank you for the comment. All signs and symptoms presented in Table 1 (first column) were reported as percentage, as observed by the symbol (%) located next to the referred comorbidity.*

10. In Table 1: you did not add percentages of comorbidities. Please add them.

*Thank you for the comment. All comorbidities presented in Table 1 (first column) were reported as percentage, as observed by the symbol (%) located next to the referred comorbidity.*

11. According to your data, the difference between females and males in mortality (32.3% vs. 30.7%) is neither statisitically or clinically signifcant. I do not understand why you insisted to say that females had higher mortality risk than males. It is not true based on your data. Please fix this accross your manuscript.

*Thank you for the comment. Indeed, the data presented in the aforementioned comment indicate that mortality was higher in females than males; however, without statistical significance. In addition, paragraph 5 of the discussion presents the hypothesis that this higher death rate in females was probably due to particularities unrelated to sex. What we discussed in the manuscript concerns survival rate and survival analysis, in which we observed that hospitalized females had a lower survival rate than males.*

12. In your limitations section, you did not reflect on your information source (eCOVID-19 platform). Is it mandatory that all hospitalized patients must be registered in this platform? If the answer is "No", then this is an obvious limitation of your dataset that makes it prone to selection bias.

*Thank you for your comment. We included information about this limitation in the last paragraph of the discussion section.*

13. Your study design can not be called "retrospective cohort study", because hisotical/retrospective cohort studies require at least two distinct groups of individuals/patients to be involved in the study from the begining. You did something like chart review not a cohort study.
You can not consider the dichtomoy of survivor vs. non-survivor as a justification for calling your study as a cohort because the ground idea of cohort designs is dividing the groups of individuals based on their exposure/predictor/independent variable not their outcome/result/dependent variable. For the outcome-based comparisons, we perform case-control studies not cohort studies.

*Thank you for the comment. The modification was made in the appropriate place in the methods section (first paragraph, page 4).*

14. Your Discussion might benefit from reflecting on hospitalization-related complications:
<https://doi.org/10.3390/jcm10040581>

*Thank you for the comment. We adjusted the discussion section to improve the manuscript and the narrative of the results.*

Sincerely,
