# Supplementary material for: Risk factors and survival in patients with COVID-19 in northeastern Brazil
Source: PLoS One. 2022 Nov 28;17(11):e0278213. doi: 10.1371/journal.pone.0278213 (PMC9704671; doi:10.1371/journal.pone.0278213)
Supplement: Attachment — Submitted filename: Response to Reviewers 2.docx [file pone.0278213.s002.docx]

We would like to thank all considerations made by the reviewers. We clarified and modified the manuscript as suggested. We also appreciate the opportunity to send this new version for peer review.

Reviewer #2: Dear authors,

Thank you for your esteemed efforts in addressing my previous comments. Unfortunately, there is a couple of critical points that were not properly addressed.

1. Study design:
Case-control studies are retrospective in nature, there is no way to conduct a case-control study prospectively. The reason for this is very simple, distribution of subjects in the study arms "cases" and "controls" is made according to presence of the outcome of interest. The group of individuals who developed the outcome are described as cases, while those who did not experience the outcome are called controls. Therefore, you can not say "retrospective case-control study".
Also, I can't see any elements of case-control design in your workflow. For example, how and from where did you select the controls? which technique did you use for matching?
Your study is more or less a chart review = retrospective analysis for patients records.

*Response: Thanks for the comment. We updated the study design section with the following: “A retrospective observational cross-sectional study was conducted.”*(page 4)

2. If you would like to use the STROBE guidelines, please make sure to follow it strictly. I can see multiple discrepancies between your manuscript structure and the STROBE checklist.

*Response: Thanks for the comment. Below is the STROBE check list (the "page" column indicates in which part of the manuscript the information can be found) used, some items were not performed by limitation or were not applicable. Because of this, it is more appropriate to remove from the text the part where the use of the STROBE checklist is mentioned (page 4)*

STROBE Statement—Checklist of items that should be included in reports of ***cross-sectional studies***

|  | Item No | Recommendation | PAGE |
| --- | --- | --- | --- |
| **Title and abstract** | 1 | (*a*) Indicate the study’s design with a commonly used term in the title or the abstract | 2 |
|  |  | (*b*) Provide in the abstract an informative and balanced summary of what was done and what was found | 2 |
|  | | |  |
| Background/rationale | 2 | Explain the scientific background and rationale for the investigation being reported | 3 |
| Objectives | 3 | State specific objectives, including any prespecified hypotheses | 3 |
| Methods | | |  |
| Study design | 4 | Present key elements of study design early in the paper | 4 |
| Setting | 5 | Describe the setting, locations, and relevant dates, including periods of recruitment, exposure, follow-up, and data collection | 4 |
| Participants | 6 | (*a*) Give the eligibility criteria, and the sources and methods of selection of participants | 4-5 |
| Variables | 7 | Clearly define all outcomes, exposures, predictors, potential confounders, and effect modifiers. Give diagnostic criteria, if applicable | 4-5 |
| Data sources/ measurement | 8* | For each variable of interest, give sources of data and details of methods of assessment (measurement). Describe comparability of assessment methods if there is more than one group | *4-5* |
| Bias | 9 | Describe any efforts to address potential sources of bias | 4-5 |
| Study size | 10 | Explain how the study size was arrived at | 6 |
| Quantitative variables | 11 | Explain how quantitative variables were handled in the analyses. If applicable, describe which groupings were chosen and why | 5 |
| Statistical methods | 12 | (*a*) Describe all statistical methods, including those used to control for confounding | 5-6 |
|  |  | (*b*) Describe any methods used to examine subgroups and interactions | 5-6 |
|  |  | (*c*) Explain how missing data were addressed | 6 |
|  |  | (*d*) If applicable, describe analytical methods taking account of sampling strategy | NA |
|  |  | (*e*) Describe any sensitivity analyses | NA |
| Results | | |  |
| Participants | 13* | (a) Report numbers of individuals at each stage of study—eg numbers potentially eligible, examined for eligibility, confirmed eligible, included in the study, completing follow-up, and analysed | 6 |
|  |  | (b) Give reasons for non-participation at each stage | 6 |
|  |  | (c) Consider use of a flow diagram | - |
| Descriptive data | 14 | (a) Give characteristics of study participants (eg demographic, clinical, social) and information on exposures and potential confounders | 6-8 |
|  |  | (b) Indicate number of participants with missing data for each variable of interest | - |
| Outcome data | 15 | Report numbers of outcome events or summary measures | 6-9 |
| Main results | 16 | (*a*) Give unadjusted estimates and, if applicable, confounder-adjusted estimates and their precision (eg, 95% confidence interval). Make clear which confounders were adjusted for and why they were included | 6-9 |
|  |  | (*b*) Report category boundaries when continuous variables were categorized | NA |
|  |  | (*c*) If relevant, consider translating estimates of relative risk into absolute risk for a meaningful time period | NA |
| Other analyses | 17 | Report other analyses done—eg analyses of subgroups and interactions, and sensitivity analyses | 5-6 |
| Discussion | | |  |
| Key results | 18 | Summarise key results with reference to study objectives | 10 |
| Limitations | 19 | Discuss limitations of the study, taking into account sources of potential bias or imprecision. Discuss both direction and magnitude of any potential bias | 13 |
| Interpretation | 20 | Give a cautious overall interpretation of results considering objectives, limitations, multiplicity of analyses, results from similar studies, and other relevant evidence | 10-14 |
| Generalisability | 21 | Discuss the generalisability (external validity) of the study results | 14 |
| Other information | | |  |
| Funding | 22 | Give the source of funding and the role of the funders for the present study and, if applicable, for the original study on which the present article is based | Journal System |

Sincerely,
