# Supplementary material for: Risk factors and survival in patients with COVID-19 in northeastern Brazil
Source: PLoS One. 2022 Nov 28;17(11):e0278213. doi: 10.1371/journal.pone.0278213 (PMC9704671; doi:10.1371/journal.pone.0278213)
Supplement: Attachment — Submitted filename: Response to reviewers.docx [file pone.0278213.s003.docx]

We would like to thank all considerations made by the reviewers. We clarified and modified the manuscript as suggested. We also appreciate the opportunity to send this new version for peer review.

Journal Requirements:

Please review your reference list to ensure that it is complete and correct. If you have cited papers that have been retracted, please include the rationale for doing so in the manuscript text, or remove these references and replace them with relevant current references. Any changes to the reference list should be mentioned in the rebuttal letter that accompanies your revised manuscript. If you need to cite a retracted article, indicate the article’s retracted status in the References list and also include a citation and full reference for the retraction notice.

*Response: Thanks for the comments. Below are the changes related to the list of references.*

1) *A correction was found in relation to reference number 8 (Zhou F, Yu T, Du R, Fan G, Liu Y, Liu Z, et al. Clinical course and risk factors for mortality of adult inpatients with COVID-19 in Wuhan, China: a retrospective cohort study. Lancet. 2020;395(10229):1054-62. doi: 10.1016/s0140-6736(20)30566-3. PMCPMC7270627). In this Article, the units for d-dimer, haemoglobin, and high-sensitivity cardiac troponin I have been corrected to μg/mL (d-dimer), g/L (haemoglobin), and pg/mL (high-sensitivity cardiac troponin I). In figure 1, the start of systematic corticosteroid for non-survivors has been changed to day 13 after illness onset. The appendix has also been corrected. These corrections have been made to the online version as of March 12, 2020, and will be made to the printed version*
2) The Original Investigation titled “Baseline Characteristics and Outcomes of 1591 Patients Infected With SARS-CoV-2 Admitted to ICUs of the Lombardy Region, Italy,” (number 13 on the reference list) published April 6, 2020, has been corrected to include the nonauthor collaborator (group) names in a supplement. The doi number remains the same as the first version.
3) *In relation to reference 17 titled “Salinas-Escudero G, Carrillo-Vega MF, Granados-Garcia V, Martinez-Valverde S, Toledano-Toledano F, Garduno-Espinosa J. A survival analysis of COVID-19 in the Mexican population. BMC Public Health. 2020;20(1):1616. doi: 10.1186/s12889-020-09721-2.” An amendment to this paper has been published and can be accessed via the original article. Because of this, was included the document (number 18 on reference list) where the correction can be found.*

 Reviewer #2:

1. Cross-sectional studies can not be described as prospective or retrospective. This is a common mistake.
2. As you said your study is cross-sectional, you do not need to say "observational".
3. The two main types of cross-sectional studies are "descriptive vs. analytical":
<https://obgyn.onlinelibrary.wiley.com/doi/full/10.1111/aogs.13331>
4. Please update the study design in your abstract accordingly.
5. The study time frame is missed.
6. I still believe that your study is better described as a "retrospective cohort" study

*Response: Thanks for the comments. After analyzing the suggested references (comment 3) and observing what is postulated in the book " Foundation of Clinical Research: Applications to practice. Portney, L.G & Watkins, M.P. Third edition. F.A Davis Company, 2015" , this research ranks more appropriately in a retrospective cohort. The necessary modification was made on page 4 and in the abstract (page 2).*

*The time frame of the study was included on page 4 (lines 2 and 3).*

Sincerely,
